# Supplementary material for: CDH-3/Cadherin, YAP-1/YAP and EGL-44/TEAD promote SYX-2/Syntaxin and EFF-1 fusogen-mediated phagosome closure
Source: bioRxiv. 2025 Apr 3:2025.04.02.646655. Preprint. [Version 1] doi: 10.1101/2025.04.02.646655 (PMC11996554; doi:10.1101/2025.04.02.646655)
Supplement: 1 — Figure S1. CCE stages and designations. TSC membrane reporter labeled in green. Wild-type CCE stages shown (a-f). (a) intact mature, (b) beading attached, (c) beading not attached, (d) soma distal retracting, (e) soma distal degrading early, (f) soma distal degrading late. Figure S2. Hyp10 phagocytic cup formation (white arrowhead) around newly formed distal node during CCE beading stage. Figure S3. Validation of CRISPR Cas9-generated GFP::SYX-2 via injury induced wound healing response at five hours post injury per published protocol (41). Wound marked with white arrowhead. [file NIHPP2025.04.02.646655V1-supplement-1.pdf]

Supplemental S1

bioRxiv preprint doi: https://doi.org/10.1101/2025.04.02.646655; this version posted April 3, 2025. The copyright holder has placed this preprint (which was not certified by peer review) in the Public Domain. It is no longer restricted by copyright. Anyone can legally share, reuse, remix, or adapt this material for any purpose without crediting the original authors.

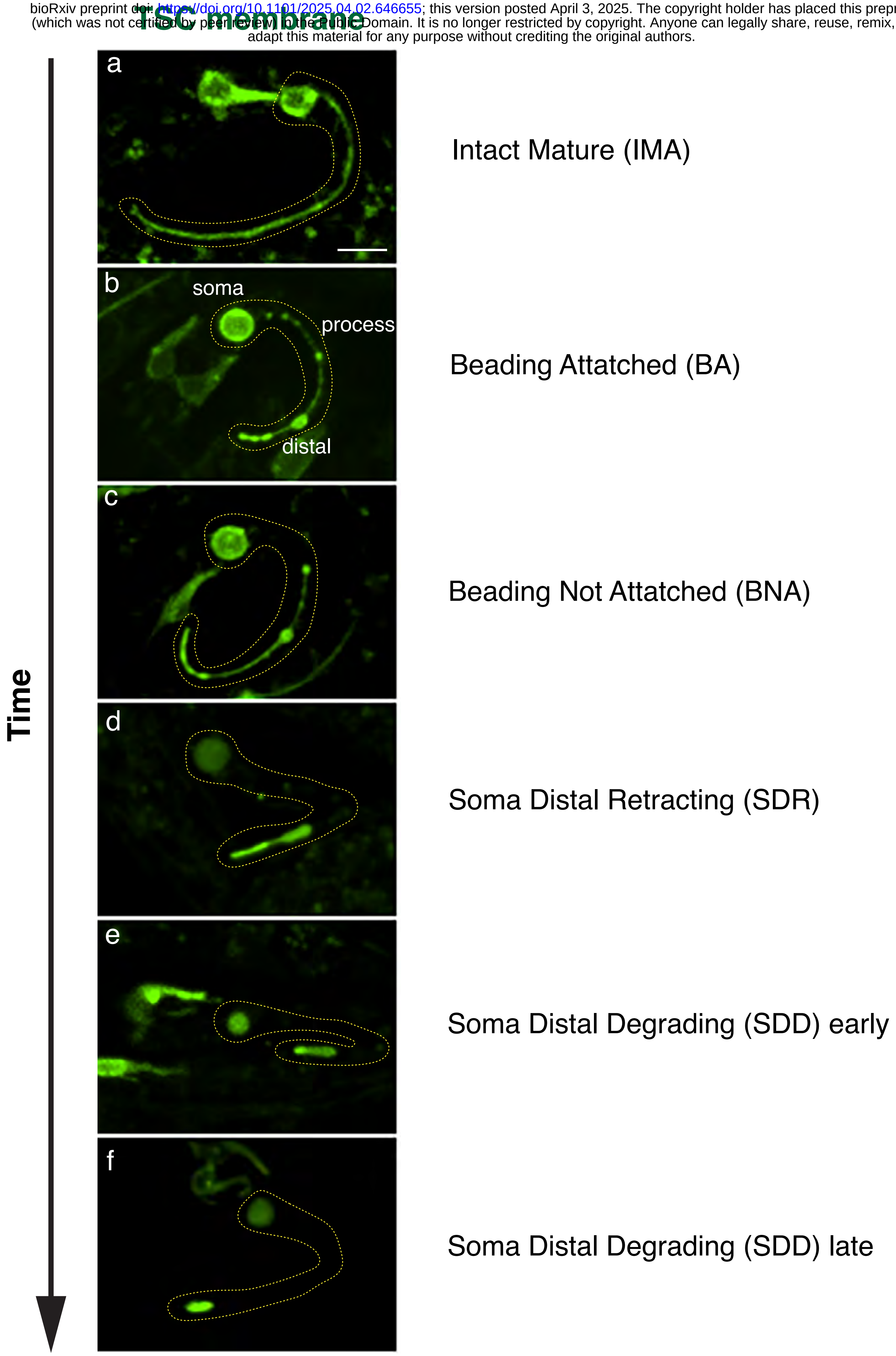

bioRxiv preprint doi: https://doi.org/10.1101/2025.04.02.646655; this version posted April 3, 2025. The copyright holder has placed this preprint (which was not certified by peer review) in the Public Domain. It is no longer restricted by copyright. Anyone can legally share, reuse, remix, or adapt this material for any purpose without crediting the original authors.

Supplemental Figure S2

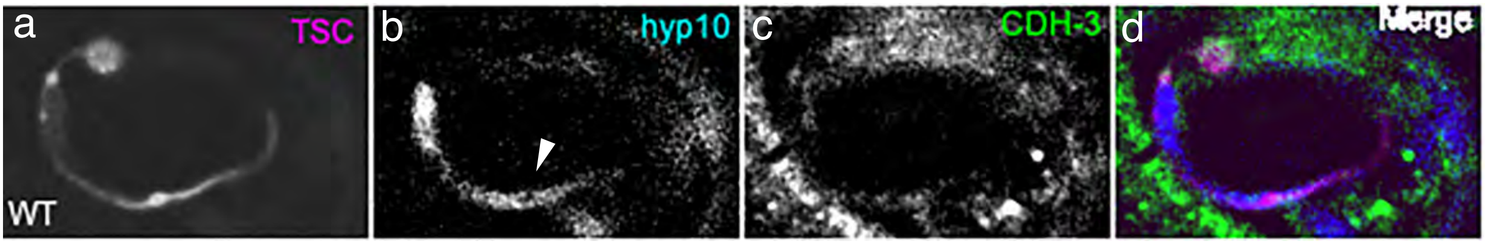

Supplemental Figure S3

bioRxiv preprint doi: https://doi.org/10.1101/2025.04.02.646655; this version posted April 3, 2025. The copyright holder has placed this preprint (which was not certified by peer review) in the Public Domain. It is no longer restricted by copyright. Anyone can legally share, reuse, remix, or adapt this material for any purpose without crediting the original authors.

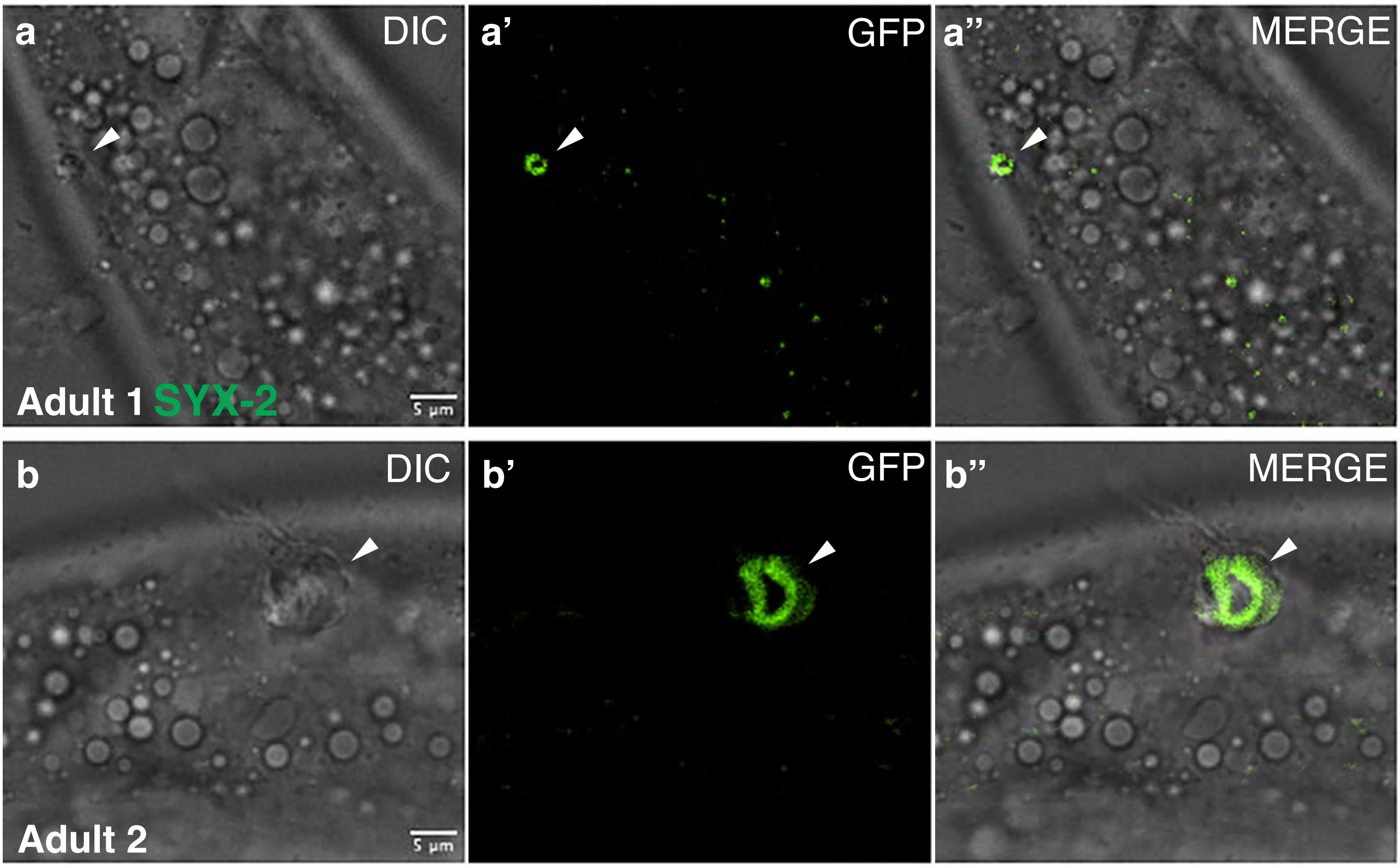

5 hours post injury
